# Jerky dystonic shoulder following infarction of the posterior thalamus

**DOI:** 10.1186/s40734-015-0022-7

**Published:** 2015-07-10

**Authors:** Ruth H. Walker

**Affiliations:** Departments of Neurology, James J. Peters Veterans Affairs Medical Center, Bronx, NY 10468 USA; Mount Sinai School of Medicine, New York City, NY USA

**Keywords:** Dystonia, Thalamus

## Abstract

The syndrome of the jerky dystonic hand is recognized as a consequence of infarction of the posterior thalamus. A patient with multiple risk factors for stroke developed jerky dystonia of more proximal involvement, affecting the shoulder and speech, several months after a stroke affecting the posterior thalamic region. The cause for the proximal, rather than distal, upper limb involvement, is unclear, and is not apparent from the distribution of the lesion on neuroimaging. Injections of botulinum toxin significantly improved the symptoms.

**Electronic supplementary material:**

The online version of this article (doi:10.1186/s40734-015-0022-7) contains supplementary material, which is available to authorized users.

## Background

The syndrome of the jerky dystonic hand is recognized as a consequence of infarction of the posterior thalamic region [1–4]. The typical nuclei involved are the ventral intermediate (Vim) and ventral caudal (Vc) nuclei [2, 4] (using Hassler’s terminology [5]), or pulvinar, ventral posterior lateral and ventral posterior medial and ventral medial nuclei, and CM-pf [3] (as per Hirai and Jones [6]). In all patients published with jerky dystonia due to cerebral infarction, the hand was affected, apart from one in whom there was proximal myoclonus of the upper limb and face but no dystonia [4].

## Case presentation

**Fig. 1:**
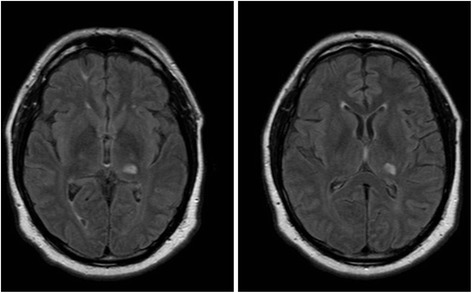
MRI brain, FLAIR sequence, shows infarction of the left posterior thalamus

A 56 year-old right-handed man presented with jerky movements affecting the right shoulder (Additional file 1). He had poorly-controlled diabetes mellitus, hypertension, and hyperlipidemia. The movements developed gradually following a stroke 4 months previously, which had presented numbness and weakness of the right limbs. Brain magnetic resonance imaging demonstrated infarction of the left Vim and Vc thalamic nuclei (Fig. 1).

On examination he had stuttering, dysarthric speech, *which appeared to be due to contractions of trunk muscles interfering with respiratory control*. Eye movements were normal. There were jerky contractions of the right shoulder muscles with neck and trunk extension. Power of all muscle groups of the right arm was 5/5 but he had difficulty voluntarily activating them. He was unable to lift the right arm voluntarily but could maintain it extended if it was lifted. He could grip with the right hand with normal strength, but was unable to release. Power was 4/5 in the right leg.

Reflexes were diminished throughout and toes were down-going. Sensation was globally diminished on the right. Standing and walking were very unsteady due to leg weakness and he used an electric scooter for mobility.

Trihexiphenidyl 5mg t.i.d. produced a moderate benefit. There was no response to amitriptyline, l-dopa/carbidopa, clonazepam, cyclobenzaprine, gabapentin, or baclofen. Injection with botulinum toxin into the right trapezius, posterior cervical muscles, and triceps resulted in a significant reduction in involuntary movements which has persisted for greater than six months. Speech was improved following injections.

## Conclusions

This patient developed an unusual jerky dystonia following a posterior thalamic infarction. Infarctions involving the Vim and ventral caudal nucleus of the thalamus generally result in jerky (“myoclonic”) dystonia involving the hand [2–4]. Electromyography would have provided a more precise interpretation of the motor disorder, i.e., whether it was true myoclonus, however, unfortunately this was not performed. Involvement of the proximal upper limb and speech has not been reported following such lesions, however, other forms of dysarthria have been reported following surgical thalamotomy for movement disorders targeting the Vim [7]. Tremor is more classically associated with the Vim nucleus of the thalamus - lesions or deep brain stimulation of this target are often used as treatment for essential tremor. Low frequency (15Hz) stimulation of Vim has been reported to cause an irregular, jerky tremor [8]. Lesioning of the Vim has been reported to improve dystonia due to infarction of the thalamic posteroventral nucleus [9].

It is not clear why this patient presented in this manner, as the lesion appears to be in a very similar location to those published (see Figure 2 in [4]), however, it may be due that the lesion affects a slightly different topographical region of the nucleus, which is not discernable with the available neuroimaging studies.

In the genetic disorder of myoclonus-dystonia (DYT11), functional imaging studies have demonstrated involvement of the ventral lateral thalamic nucleus [10] and posterior thalamus [11]. Deep brain stimulation of the Vim may be a potential therapeutic intervention in this disorder (although globus pallidus pars interna may be a superior target) [12]. Myoclonus has been reported following posterior thalamic hematoma [13], at a very similar location to that in our patient. As this site is the target of cerebello-thalamic inputs, it is possible that this phenomenology supports a role for the cerebellum in dystonia (e.g. [14, 15] and reviewed in [16]), although this remains controversial.

## Consent

Written informed consent was obtained from the patient for publication of this Case report and any accompanying images. A copy of the written consent is available for review by the Editor-in-Chief of this journal.

## Additional file

Additional file 1:
**The patient has frequent jerky, repetitive contractions of the right shoulder musculature, with involvement of the right triceps, and extension of the neck and trunk.**
